# The gut microbiome in cardio-metabolic health

**DOI:** 10.1186/s13073-015-0157-z

**Published:** 2015-03-31

**Authors:** Tue H Hansen, Rikke J Gøbel, Torben Hansen, Oluf Pedersen

**Affiliations:** The Novo Nordisk Foundation Center for Basic Metabolic Research, Section of Metabolic Genetics, Faculty of Health and Medical Sciences, University of Copenhagen, Universitetsparken 1, København Ø, 2100 Denmark; Faculty of Health Sciences, University of Southern Denmark, J.B. Winsløws Vej 19-3, Odense C, 5000 Denmark

## Abstract

With the prevalence of cardio-metabolic disorders reaching pandemic proportions, the search for modifiable causative factors has intensified. One such potential factor is the vast microbial community inhabiting the human gastrointestinal tract, the gut microbiota. For the past decade evidence has accumulated showing the association of distinct changes in gut microbiota composition and function with obesity, type 2 diabetes and cardiovascular disease. Although causality in humans and the pathophysiological mechanisms involved have yet to be decisively established, several studies have demonstrated that the gut microbiota, as an environmental factor influencing the metabolic state of the host, is readily modifiable through a variety of interventions. In this review we provide an overview of the development of the gut microbiome and its compositional and functional changes in relation to cardio-metabolic disorders, and give an update on recent progress in how this could be exploited in microbiota-based therapeutics.

**Electronic supplementary material:**

The online version of this article (doi:10.1186/s13073-015-0157-z) contains supplementary material, which is available to authorized users.

## Introduction

The dramatic increase in the prevalence of obesity worldwide [1,2] is associated with serious health risks, including increased susceptibility to co-morbidities such as type 2 diabetes (T2D) and cardiovascular disease (CVD). Both genetic susceptibility and environmental factors (such as intrauterine conditions, physical inactivity, smoking and unhealthy dietary habits) are involved in the pathogenesis of cardio-metabolic disorders [3,4]. Recent findings suggest that the environmental component in the development of metabolic dysfunction is mediated, at least in part, through an altered gut microbial structure and function. The gut microbiota maintains several essential functions, including colonic fermentation of dietary fibers; extraction of nutrients; synthesis of certain vitamins; prevention against colonization by pathogens; maturation of the intestinal epithelium and immune system; release of metabolites to the systemic tissues; and modulation of gastrointestinal hormone release and nerve function [5-8]. It is thus increasingly recognized that the configuration and activity of the gut microbiota, influenced by host genotype and additional environmental factors, affect host biology in numerous ways.

Recent studies in animal models and humans have found correlations between specific alterations in the gut microbiota community structure and the onset of diabetes, obesity and CVD. There is evidence for the influence of diet on the gut microbiota in various diseases, and several microbial metabolites have been shown to have a significant effect on the metabolic health of the host through pathways that influence intestinal health, such as mucosal permeability, inflammation and immune function [9-11]. Lifestyle-related shifts in the bacterial composition and function can break the delicate balance of the microbial composition and lead to a state of chronic disequilibrium referred to as dysbiosis, a condition that has been associated with several pathologies, including obesity and T2D [12-17].

In this review, we examine what current technologies have taught us about the origins and composition of the gut microbiome, and how this is associated with cardio-metabolic disease. We consider recent studies that provide insights into the role of the human gut microbiota in the metabolic health spectrum, and discuss how this relationship can be exploited and modulated to improve health.

## Gut microbiota composition

**Figure 1:**
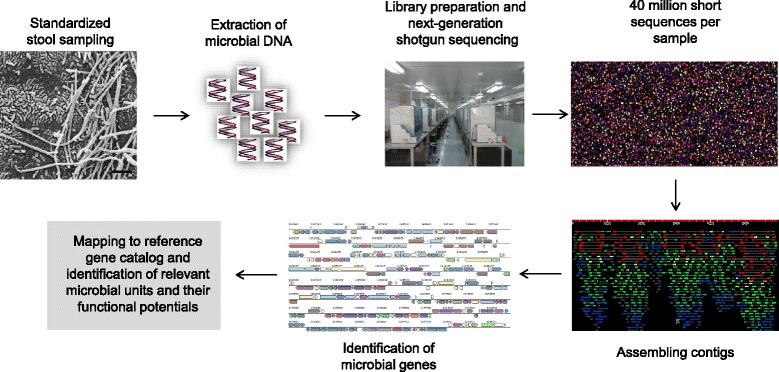
**Example of a quantitative metagenomics pipeline.** A metagenomics pipeline involves a series of sequential and crucial steps. Firstly, sampling of the relevant biological material and subsequent extraction of DNA are conducted in a standardized manner to ensure good-quality DNA in high nanogram or microgram quantity. Secondly, a library is prepared by fusing DNA fragments with adapter molecules followed by PCR amplification and next-generation sequencing, which produces millions of short reads (about 150 to 800 bp depending on the sequencing platform) that are then assembled into longer contigs. Finally, microbial genes are identified and annotated to known functions or taxonomic units based on homology searches against available reference catalogs. For further details see [166].

Culture-independent methods are now being used to analyze microbial ecosystems, combining high-throughput sequencing of nucleic acids (DNA, RNA) with advanced bioinformatics for taxonomic mapping. Before these approaches, only those microbes that could be cultured were characterized, comprising about 20% of the total [18]. Because the majority of intestinal bacterial species are anaerobic and difficult to cultivate, gene-based studies have now been developed to study the broad spectrum of both anaerobic and aerobic gut bacteria. These technologies include targeted rRNA gene markers such as 16S rRNA gene sequencing, which focus on microbial composition and diversity, and untargeted shotgun sequencing, which in addition enables assessment of the functional capability of the gut microbiota (Box 1 and Figure 1) [19].

As a result of the application of these technologies, much has been learned about the origins and composition of the human microbiome in the past 10 years. Although the human gut is not entirely sterile *in utero* [27,28], the extensive colonization of the gastrointestinal tract begins at birth through the transfer of bacteria from the vagina, anus and skin of the mother. In this way the newborn gut is colonized by facultative anaerobic bacteria, such as Enterobacteriaceae, streptococci and staphylococci, and these bacterial communities are highly variable in the early stage [29]. The specific bacterial pattern differs in children delivered vaginally or by Cesarean section, with the latter affecting microbiota composition in both infancy [30] and childhood [31] and being associated with a reduced bacterial diversity [32]. This offers a potential explanation for the increased risk of atopic disorders [33] and obesity [34] observed in children delivered by Cesarean section. In addition, the gut colonization pattern differs between full-term and preterm infants [35]. The immature preterm gut along with prolonged admission at neonatal care units and the possible use of broad spectrum antibiotics delay the establishment of a beneficial bacterial community and enable the growth of potentially pathogenic bacteria [35].

Besides mode of delivery and gestational age, neonatal diet is a determining factor, and *Escherichia coli* and streptococci are the first bacteria to appear in newborn breast-fed infants, followed by bifidobacteria, which quickly become predominant. The microbiota of formula-fed infants shows even higher variability and diversity, having lower counts of bifidobacteria and higher counts of clostridia and enterococci [29]. Weaning and the transition to solid foods confers a compositional change towards a more adult-like microbiota dominated by *Clostridium* spp. and *Bacteroides* spp. [36], with a stable adult-like microbiota, often referred to as ‘the core microbiome’ [37], being established at approximately the age of 3 years [38]. Most bacterial species in the adult human gut belong to the phyla Firmicutes (about 60%), Bacteroidetes (about 15%) and Actinobacteria (about 15%), but less abundant microbial phyla, such as Verrucomicrobia (about 2%), Proteobacteria (about 1%) and Methanobacteriales (archaea) (about 1%), are also represented [24,39-41]. Despite the phylum-level similarities, the species-level composition is highly variable between individuals, caused by environmental influence and genetic predisposition [42,43]. Contrary to the high inter-individual variation [44,45], with up to 2,187-fold variability of microbial abundance at the species level [24], the structure of the gut microbiota of an individual remains relatively stable over time in the absence of perturbation [46,47], until advanced age when the microbiota becomes less diverse and more unstable, probably related to alteration in immune competencies [48].

## The association of gut microbiota with cardio-metabolic disorders

The prevalence of cardio-metabolic disorders (obesity, T2D and CVD) is increasing globally and has for several years been the number one cause of death worldwide [49]. Within the past decade the role of the gut microbiota in metabolic disorders has been extensively investigated (Table 1), and accumulating evidence indicates that a state of bacterial dysbiosis contributes to differences in body weight, insulin sensitivity, glucose metabolism and other cardio-metabolic risk factors among individuals [50], strengthening the gut microbiota as a potential risk factor in the pathogenesis of obesity and its co-morbidities. Here, we look at the main cardio-metabolic traits and their associations with gut microbiota composition.

**Table 1:** **Human metagenomic studies of the gut microbiome and cardio-metabolic traits**

**Study**	**Aim**	**Number of participants**	**Design**	**Method**	**Main findings**
Ley *et al.* (2006) [41]	To investigate the effect of either a carbohydrate- or fat-restricted diet on gut microbial ecology	14 adults; 12 obese, 2 lean	Randomized intervention	16S rRNA	• Increased abundance of Firmicutes and reduced abundance of Bacteroidetes in obese
					• Increased abundance of Bacteroidetes and decreased abundance of Firmicutes following 1 year of either fat- or carbohydrate-restricted low-calorie diet
					• Increase in Bacteroidetes correlated with body weight reduction regardless of diet
Zhang *et al.* (2009) [161]	To investigate microbiota composition in morbid obesity and following RYGB	9 adults; 3 lean, 3 morbidly obese, 3 post RYGB	Cross-sectional	16S rRNA, qPCR	• Firmicutes dominant in normal weight and obese, decreased in post RYGB
					• Gamma-Proteobacteria increased whereas *Clostridia* decreased post-RYGB
					• *Prevotellaceae* highly enriched in obese
					• Methanobacteriales highly abundant in obese, while non-detectable in normal weight
Turnbaugh *et al.* (2009) [37]	To investigate the influence of host genotype, environmental exposure and host adiposity	154 adults; 31 MZ twin pairs, 23 DZ twin pairs, (concordant for obesity or leanness), 46 mothers	Cross-sectional	16S rRNA	• Lower proportion of Bacteroidetes and a higher proportion of Actinobacteria in obese
					• Obesity associated with reduced diversity
					• Obese microbiome enriched for genes involved in macronutrient metabolism
Larsen *et al.* (2010) [12]	To investigate differences in gut microbiota composition associated with T2D	20 adults; 10 T2D, 10 NGT	Cross-sectional	16S rRNA, qPCR	• Decreased diversity in T2D
					• Firmicutes, including Clostridia, decreased in T2D
					• The ratio of the phylogenetic groups *Bacteroides-Prevotella* to *Clostridium coccoides-Eubacterium rectale* and the Bacteroidetes to Firmicutes ratio correlated positively with 2 h p-glucose during an OGTT
					• Beta-Proteobacteria highly enriched in T2D and correlated with 2 hour p-glucose during an OGTT
Jumpertz *et al.* (2011) [5]	To investigate the effect of caloric intake on microbiota composition in lean and obese	21 adults; 12 lean, 9 obese	Randomized cross-over intervention	16S rRNA	• High-calorie diet changes the relative abundance of microbiota on the phylum (Bacteroidetes versus Firmicutes), class (Bacteroidetes versus Clostridia), and order level (Bacteroidales versus Clostridiales)
					• Phylum-, class-, and order-level changes in microbiota composition during intervention associated with fecal caloric content in lean but not in obese
Koren *et al.* (2011) [59]	To investigate the bacterial diversity of atherosclerotic plaque, oral cavity and gut in patients with CVD	30 adults; 15 CVD, 15 healthy	Cross-sectional	16S rRNA, qPCR	• No phylum- or genus level compositional difference between CVD patients and healthy controls
					• Several shared OTUs between atherosclerotic plaque and fecal samples
Karlsson *et al.* (2012) [60]	To investigate the microbiota composition in patients with CVD	25 adults; 13 CVD, 12 healthy controls	Cross-sectional	Quantitative metagenomics	• *Colinsella* enriched in patients, *Eubacterium* and *Roseburia* enriched in controls
					• Genera of Clostridiales, *Clostridium* and *Peptostreptococcus* negatively correlated with hsCRP
					• Atherosclerosis associated with the *Ruminococcus* enterotype
Qin *et al.* (2012) [13]	To investigate differences in gut microbiota composition and function associated with T2D	368 adults; 183 T2D cases, 185 healthy controls	Cross-sectional	Quantitative metagenomics	• T2D associated with moderate dysbiosis with a decline in butyrate-producing bacteria
					• Gut-microbiome-based T2D index accurately classifies T2D individuals
Le Chatelier *et al.* (2013) [55]	To investigate the bacterial abundance in lean and obese	292 adults; 123 lean, 169 obese	Cross-sectional/retrospective	Quantitative metagenomics and 16S rRNA	• Low bacterial richness associates with increased overall adiposity, insulin resistance, dyslipidemia, and a more pronounced inflammatory phenotype
					• Discrimination between high versus low gene count and obesity status possible from a combination of only four species with ROC analysis AUC of 0.97
					• Increased weight gain in individuals with low microbial gene count
Karlsson *et al.* (2013) [16]	To investigate differences in gut microbiota composition and function associated with T2D	145 adults; 53 T2D, 49 IGT, 43 NGT	Cross-sectional	Quantitative metagenomics	• Increased abundance of *Lactobacillus* spp. and abundance of *Clostridium* spp. decreased
					• *Clostridium* spp. correlated with fasting glucose and HbA1c, whereas *Lactobacillus* spp. correlated negatively with fasting glucose, insulin, C-peptide and TAG, and positively with adiponectin and HDL
					• Microbiota composition as determined by metagenomic clusters better correlated with T2D than known clinical risk factors (WC, WHR and BMI)
Zhang *et al.* (2013) [17]	To investigate differences in gut microbiota composition associated with T2D	121 adults; 44 NGT, 64 IGT, 13 T2D	Cross-sectional	16S rRNA	• Higher abundance of Clostridia in T2D
					• Negative trend of abundance of *Streptococcus* from NGT to IGT to T2D
					• Enterotype classification not associated with glucose tolerance status
					• 28 OTUs associated with glucose tolerance status
					• Fasting glucose associated with microbiota composition
					• Fasting insulin inversely associated with alpha (intraindividual) diversity
Kong *et al.* (2013) [160]	To investigate the impact of RYGB on microbiota composition	30 adults; 7 T2D, 23 non-T2D obese	Non-randomized intervention	16S rRNA	• Increased bacterial richness following RYGB, mainly within the phylum Proteobacteria
					• RYGB induced genus-level changes in microbiota composition correlated with changes in white adipose tissue gene expression
Graessler *et al.* (2013) [14]	To investigate the impact of RYGB on microbiota composition and function	6 adults; 5 T2D, 1 non-T2D obese	Non-randomized intervention	Quantitative metagenomics	• Relative abundance of 22 species an 11 genera affected 3 months after RYGB
					• Overall, RYGB induced phylum-level changes characterized by reduction in Bacteroidetes and Firmicutes and an increase in Proteobacteria and Verrucomicrobia
					• Species-level changes dominated by an increase in *A. muciniphila*, *E. coli* and *K. pneumonia* and a decrease in *F. prausnitzii*, *E. rectale* and *D. invisus.*

*Abbreviations:* *AUC* area under the curve, *BMI* body mass index, *CVD* cardiovascular disease, *DZ* dizygotic, *HDL* high-density lipoprotein, *hsCRP* high-sensitivity C-reactive protein, *IGT* impaired glucose tolerance, *MZ* monozygotic, *NGT* normal glucose tolerance, *OGTT* oral glucose tolerance test, *OTU* operational taxonomic unit, *qPCR* quantitative PCR, *ROC* receiver operating characteristic, *RYGB* Roux-en-Y gastric bypass, *TAG* triacylglyceride, *T2D* type 2 diabetes, *WC* waist circumference, *WHR* waist-hip ratio.

### Obesity

Evidence from several studies in humans and rodents points to the gut microbiota as a contributing factor in obesity development. Studies of the gut microbiota in mice genetically predisposed to obesity (*ob/ob*) have shown a decreased Bacteroidetes-to-Firmicutes ratio [39], which is associated with an increased capacity for energy harvest from otherwise indigestible dietary polysaccharides [51]. Studies of germ-free mice have demonstrated that they are leaner and more resistant to diet-induced obesity when introduced to a high-fat western-style diet than their conventional counterparts [52,53]. After colonizing germ-free mice with microbiota from normal mice, the amount of body fat increased and the insulin sensitivity decreased [52]. Similarly, when colonizing germ-free mice with fecal microbiota from human twin pairs discordant for obesity, mice receiving microbiota from the obese twin showed more weight gain and adiposity than those receiving microbiota from the lean sibling, hinting at a causal effect for the transplanted microbial community [54]. Although inconsistent findings at the phylum level have been reported in human obesity, reduced bacterial diversity has been associated with adiposity [37]. In the Metagenomics of the Human Intestinal Tract (MetaHIT) study including 292 non-diabetic Danish individuals, 23% of the studied population sample had low microbial gene richness, with the bacterial gene count reduced by 40% [55]. Interestingly, individuals with low richness were characterized by low-grade inflammation, insulin resistance, dyslipidemia and overall adiposity, which potentially put them at an increased risk of progression to obesity-related co-morbidities.

### Type 2 diabetes

It has been reported that patients with T2D have a different taxonomic composition and functional potential of their gut microbiome from that of non-diabetic individuals [12-17]. One recent 16S rRNA-based study observed a positive correlation between fecal bifidobacteria and plasma insulin and insulin resistance [56], in disagreement with the previously detected inverse relationship found in mice [57] and humans [58]. A metagenomics-based study including 344 Chinese individuals identified a moderate degree of gut bacterial dysbiosis, with a decline in butyrate-producing bacteria and an increase in opportunistic bacteria among T2D patients [13]. However, no difference in diversity between T2D patients and healthy controls was observed. A study of 145 Swedish women with normal, impaired or diabetic glucose regulation found comparable, but not identical, compositional changes associated with T2D, suggesting ethnic or regional influences [16]. In both the Swedish and the Chinese study, it was possible to discriminate with high accuracy between T2D patients and non-diabetic controls with few metagenomic markers [13,16]. Despite minor discrepancies between individual studies, evidence is mounting that T2D is associated with both functional and compositional changes of the gut microbiota. However, it remains to be firmly established whether these changes are causal or secondary to physiological changes or perhaps pharmacologically induced.

### Atherosclerosis and cardiovascular disease

Thus far, evidence directly linking specific changes in the human gut microbiota with CVD has been limited to a few smaller cross-sectional studies. Although failing to show any overall structural difference between cases and controls on either the phylum or the genus level, a study of 15 patients with clinical atherosclerosis and an equal number of healthy controls matched for sex and age showed several shared operational taxonomic units (OTUs) between atherosclerotic plaques and fecal samples, suggesting that plaque microbiota may at least partially be of intestinal origin [59]. Further analyses using whole-genome sequencing showed that the genus *Collinsella* was enriched in fecal samples from patients with atherosclerosis, whereas *Roseburia* and *Eubacterium* were enriched in healthy controls [60]. In the same study, Clostridiales genera *Clostridium* and *Peptostreptococcus* were negatively correlated with high-sensitivity C-reactive protein, an inflammatory marker strongly associated with increased risk of CVD [61].

## Mechanisms of microbiota-host interactions

**Figure 2:**
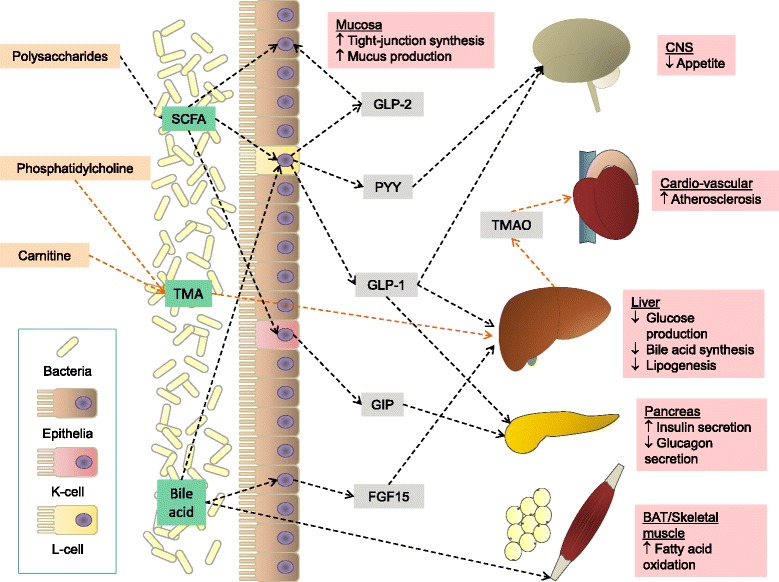
**Gut microbiota-host interactions.** The short-chain fatty acids (SCFA) propionate, acetate and butyrate produced by bacterial fermentation of indigestible polysaccharides trigger the release of the satietogenic gut hormones GLP-1 and PYY from enteroendocrine L-cells; these hormones in turn regulate ingestive behavior by acting on the hypothalamus. Release of gastric inhibitory polypeptide (GIP) from enteroendocrine K-cells triggered by butyrate is a potent promoter of glucose-dependent insulin secretion, acting in concert with GLP-1. Through direct trophic effects on the intestinal epithelium and by triggering the release of GLP-2 from L-cells, butyrate makes the epithelial barrier less permeable through increased mucus production and tight junction expression. L-carnitine and phosphatidylcholine, both constituents of red meat, are metabolized by intestinal bacteria, releasing trimethylamine (TMA). Following absorption to the portal circulation, TMA is converted by hepatic flavin-containing monooxygenase to the atherogenic trimethylamine-*N*-oxide (TMAO). The gut microbiota is heavily involved in bile acid metabolism by performing deconjugation and dehydroxylation. Cholic acid lowers hepatic lipogenesis by acting on the farnesoid X receptor and increases the energy expenditure through fat oxidation by inducing the enzymatic conversion of inactive thyroxine to the active tri-iodothyronine in brown adipose tissue (BAT) and skeletal muscle. Abbreviations: CNS, central nervous system.

Although convincing evidence suggests that a dysbiotic state of the gut microbiota composition is associated with metabolic disease, we are still far from fully understanding the complexities of the multiple underlying mechanisms, as outlined below and summarized in Figure 2.

Studies of mice provide early evidence that the gut microbiota has an essential role in energy harvest, in that germ-free mice need to consume approximately 30% more calories to sustain a body weight similar to conventionally raised mice [52]. An essential mechanism by which the gut microbiota enhances energy harvest is through the hydrolysis and fermentation of otherwise indigestible dietary polysaccharides. Microbial fermentation generates monosaccharides and short chain fatty acids (SCFAs), which can be absorbed and used as energy by the host [62]. Of the three main SCFAs, propionate, acetate and butyrate, the latter in particular has attracted attention as a potential mediator of the metabolic effects of dysbiosis (reviewed by Brahe *et al.* [63]), with several studies in humans demonstrating a reduced proportion of butyrate-producing Clostridiales in T2D and CVD [13,16,60]. Butyrate acts by diverse molecular mechanisms to regulate energy intake, expenditure and storage [64,65]. It stimulates the release of the satietogenic hormones glucagon-like peptide 1 (GLP-1) and peptide YY (PYY) from enteroendocrine L-cells, at least in part by acting as an agonist for the free fatty acid receptors 2 and 3 (FFAR2/3) [66], thus regulating the ingestive behavior of the host. Similarly, by stimulating the release of gastric inhibitory polypeptide (GIP) from enteroendocrine K-cells it potentially increases host insulin sensitivity in concert with GLP-1 [67]. Butyrate also strengthens the integrity of the intestinal epithelial barrier through effects on mucus production [68] and tight junction expression [69], as well as conferring trophic effects on the epithelial cells, possibly mediated by an incretin hormone, glucagon-like peptide 2 (GLP-2) [70]. The overall effect of this is a reduced passage of toxic and pro-inflammatory substances across the epithelial barrier. Interestingly, the presence of bacteria and bacterial residues such as lipopolysaccharide in host blood and other tissues is linked to low grade inflammation, obesity and T2D [71-74], emphasizing the importance of a sustained intestinal barrier in relation to metabolic disorders.

Another way that the gut microbiota influences the metabolic state of the host is through an extensive involvement in bile acid homeostasis [75]. Direct evidence from rodent studies shows that treatment with broad-spectrum antibiotics (for example, ampicillin) increases bile acid reabsorption through increased expression of the transporter SLC10A2 [76], while simultaneously increasing hepatic bile acid synthesis by suppressing the expression of fibroblast growth factor 15 [77]. These findings provide clues to the molecular mechanisms underlying the decades-old findings that germ-free animals have substantially higher bile acid concentration in bile [78] along with decreased bile acid excretion in feces [79]. A more recent study gives further weight to the importance of the gut microbiota in bile acid homeostasis; this [80] showed that the mouse gut microbiota regulates expression of cholesterol 7-alpha hydroxylase, the rate-limiting enzyme in bile acid synthesis, through deconjugation of tauro-conjugated muricholic acid, a potent antagonist of the farnesoid X receptor (FXR). Acting on the same receptor, cholic acid reduces circulating triglyceride levels by decreasing the hepatic expression of the SREBP-1c transcription factor controlling genes involved in lipogenesis [81]. Interestingly, FXR deficiency has been reported to improve glucose homeostasis in obese mice [82]. In another study in mice, supplementation with cholic acid protected against and even reversed diet-induced obesity; the effect was mediated by increased expression of the type 2 iodothyronine deiodinase (D2) enzyme and thus increased conversion of inactive thyroxine (T4) to the active tri-iodothyronine (T3) in brown adipose tissue, effectively raising energy expenditure through fat oxidation [83]. Similar effects, regulated by the cAMP-protein kinase A pathway by activation of the G-protein-coupled receptor TGR5, were demonstrated in human skeletal muscle *in vitro* [83]. Stimulation of the TGR5 signaling pathway by bile acids has also been demonstrated to induce GLP-1 release from enteroendocrine L-cells and consequently improve glycemic regulation in obese mice [84]. These discoveries have spurred an interest in bile acids as potential therapeutic targets in metabolic disorders. Interestingly, colestimide, a bile acid sequestrant, decreases body weight and improves glucose tolerance and insulin sensitivity in diet-induced obese mice [85], and it improves glycemic regulation and reduces central adiposity in T2D humans and mice [86,87].

Although the evidence for an association between gut microbiota and CVD at the compositional level is scarce, at the functional level metabolism of specific dietary components by the gut microbiota, particularly trimethylamine-containing compounds such as phosphatidylcholine (PC) and carnitine, has been linked to development of CVD. PC- and carnitine-rich foods include red meats, fish, poultry, dairy products and eggs [88,89]. Following ingestion both compounds are metabolized by the gut microbiota to trimethylamine (in the case of carnitine, with γ-butyrobetaine as an intermediary metabolite), which then enters the circulation and is converted by hepatic flavin-containing monooxygenase to trimethylamine-*N*-oxide (TMAO) [90,91]. Studies have shown that TMAO was undetectable in germ-free mice following a carnitine or choline challenge, but following colonization of the animals with microbiota from conventional mice, they gained the ability to produce TMAO [92,93]. Furthermore, antibiotic treatment in conventional mice reduced TMAO production after a choline or carnitine challenge [92,93], with similar effects reported in humans [93,94]. Mice genetically deficient in phosphatidylethanolamine-*N*-methyltransferase, an enzyme involved in hepatic PC synthesis, are protected from developing atherosclerosis [95], whereas dietary supplementation with either carnitine, γ-butyrobetaine, choline or TMAO increases atherosclerotic plaque formation in mice genetically susceptible to atherosclerosis [91-93]. In addition, diet-induced TMAO production and atherosclerosis susceptibility is transmissible through microbiota transplantation [96]. It is thus well established that the gut microbiota is intrinsically involved in TMAO production and that TMAO, rather than its precursors choline and carnitine, is the pro-atherogenic compound.

Although the way in which TMAO promotes the formation of atherosclerotic plaques is not entirely clear, several contributing mechanisms have been identified. TMAO has been show to inhibit reverse cholesterol transport by reducing the expression of the hepatic enzymes Cyp7a1 and Cyp27a1, as well as multiple bile acid transporters, while simultaneously inhibiting the expression of intestinal bile acid transporters Npc1L1 and Abcg5/8, thus reducing bile acid synthesis and excretion by the liver and bile acid uptake in the intestine [93]. In macrophages TMAO increases the efflux of cholesterol through increased expression of the cholesterol transporters Abca1 and Abcg1 while simultaneously increasing expression of the proatherogenic scavenger receptors, CD36 and scavenger receptor A [92,93]. In recent years several human clinical studies have demonstrated a clear association between trimethylamine-containing compounds and CVD risk (Table S1 in Additional file 1). Plasma choline level is a strong predictor of major cardiac events in patients with suspected acute coronary syndrome [97], as well as in individuals with stable coronary artery disease; interestingly, in the latter disease it is a predictor only when there are concomitant increased TMAO levels [98]. Similarly, carnitine is an independent predictor of prevalent CVD and incident major cardiac events, but only with concurrent high TMAO levels [93]. TMAO in itself is also an independent predictor of incident death, myocardial infarction and stroke, as well as prevalent CVD even in individuals at low risk of cardiovascular events [94]. Interestingly, elevated TMAO levels have also been reported in patients with T2D [99], chronic kidney disease [100] and heart failure [99,101], disorders that are known to be associated with an increased risk of CVD.

## Modulation of the gut microbiota for prevention and therapy

With the establishment of the gut microbiota as an environmental factor contributing to the development of cardio-metabolic disorders, interest in modulating the microbiota composition and function as a therapeutic strategy is increasing. Theoretically, mechanisms through which modulation of the gut microbiota might be achieved include lifestyle changes, such as dietary intervention or exercise, surgery or the use of pharmaceuticals, or through transplantation from healthy donors.

### Diet

Variation in diet composition is a major contributing factor in determining microbiota diversity. Studies comparing the diversity and phylogenetic composition of gut microbiota from individuals consuming a western-type diet (rich in fat and animal protein) and indigenous people from Africa and South America (living on a diet dominated by plant-based polysaccharides) have demonstrated that the former is associated with an increase in the abundance of *Bacteroides* spp., whereas the latter is associated with increased *Prevotella* spp. and overall diversity [38,102]. At the phylum level, studies of the effect of high-fat diet in murine models have shown a decrease in the ratio of Bacteroidetes to Firmicutes [103-106], although human studies have not been equally consistent [11,41,107]. It is clear, however, that composition and functional capabilities of the human gut microbiota rapidly adapt to changes in macronutrient content of the diet [9-11], mirroring studies in mice that have demonstrated that dietary changes result in an adaptation of the gut microbiota within a few days [104,105,108]. Still, compositional changes following dietary intervention are subtle and readily reversible [109,110], and although there are indications that a permanent change in diet could lead to persistent effects on the structure of the gut microbiota [48], the long-term effect of dietary intervention in humans needs to be investigated further. However, recent studies in humans and rodents have demonstrated a remarkable ability of the metabolic capacity of the gut microbiota to be altered by long-term dietary patterns [91,93], indicating that the potential benefits of dietary intervention on host metabolism might very well be significant.

### Prebiotics

The term prebiotics refers to dietary components (mainly non-digestible oligosaccharides) that selectively stimulate the growth or activity of a limited number of microbial genera or species [111]. Studies have consistently shown that the gut microbiota can be modulated by administration of inulin-type fructans and galactans, with a preferential effect on *Bifidobacteria* and to some extent *Lactobacillus* spp. (reviewed by Meyer and Stasse-Wolthuis [112]). In a series of studies in rodents and humans, prebiotics acting through changes in microbiota composition have been shown to reduce energy intake and body weight [113,114], concomitantly reducing insulin resistance and hyperglycemia [115-117]. These effects appear to be mediated by increased release of the anorexigenic gut hormones GLP-1, GLP-2 and PYY [70,113,114,116,118], by reduced release of the orexigenic peptide ghrelin [114,118-120], and by reducing endotoxemia by way of improved mucosal barrier function, thus reducing levels of inflammatory markers [57,117,121-123]. Directly affecting the production of SCFAs (butyrate) is another potential mechanism by which prebiotics might beneficially influence host physiology [124-126].

### Probiotics

Probiotics are live microorganisms that, when ingested in sufficient amounts, exert beneficial health effects on the host. The majority of known bacterial probiotics belong to the Gram-positive genera of *Lactobacillus* and *Bifidobacterium*, with the Nissle 1917 strain of *E. coli* being the only extensively investigated Gram-negative probiotic. The therapeutic effects of probiotics in metabolic disorders have been heavily investigated for the past decade, with promising results, although mainly in murine models. Several studies in such models of obesity and diabetes have demonstrated an improved metabolic profile (including but not limited to reduced levels of glucose, insulin, HbA1c, LDL-cholesterol, triglyceride and leptin) following administration of different strains of lactobacilli and bifidobacteria [127-137]. Although human trials have been less consistent (Table S2 in Additional file 1), the majority of studies show a beneficial metabolic effect of probiotics, with only a few smaller studies failing to show an improvement in cardio-metabolic variables.

Increasing knowledge about microbiota-host interactions of specific bacterial species and their metabolites has sparked an interest in so-called next-generation probiotics: the use of live genetically modified bacteria to deliver therapeutic molecules to the gut for further processing by the commensal bacterial flora or direct absorption by the host. In a recent study, Chen *et al.* [138] demonstrated that administering *E. coli* genetically modified to produce *N*-acyl-phosphatidylethanolamines (NAPE), the precursors for the potent anorexigenic *N*-acylethanolamides (NAE), to high-fat fed obese mice for a total of 8 weeks resulted in reduced weight gain and adiposity, without any difference in lean body mass from controls. These effects on body composition endured for 4 weeks after cessation of bacterial administration, and even after 12 weeks the treated mice weighed significantly less. Treated mice also had a more favorable metabolic profile, with lower levels of circulating leptin and insulin, and improved glucose tolerance and insulin sensitivity, along with reduced hepatic steatosis and inflammation. Although treated mice had markedly lower food intake and higher energy expenditure, the latter as a consequence of increased basic metabolic rate, the results indicated a direct effect of NAE at the transcriptional level, with increased expression of genes involved in fatty acid oxidation. Clinical trials of recombinant bacteria in relation to cardio-metabolic disorders have yet to be performed, but the feasibility in humans has been demonstrated in patients with inflammatory bowel disease [139].

### Physical activity

Decreased levels of physical activity is a well-established factor contributing to the global obesity epidemic [140]. Evidence for the existence of a modulating effect of physical activity on the gut microbiota is accumulating, although mainly from rodent models. Physical activity has been shown to affect gut microbiota composition and diversity in healthy rats [141,142], as well as in murine models of obesity [143,144] and hypertension [144], with some results indicating that the demonstrated effects are independent of diet [143,145]. So far, studies in humans are few and limited in design, with only one smaller study showing increased diversity and structural changes on the family and genus level in athletes [146]. It is, however, unclear to what extent these changes are attributable to concomitant dietary differences rather than physical activity alone, and future studies are needed to discriminate between these effects and to clarify potential mediating factors.

### Antibiotics

Administration of antimicrobial agents, including broad-spectrum antibiotics, has been proposed as a possible contributor to the obesity epidemic and the shrinking gut microbiome richness in the western world. This theory is supported by a few, but large epidemiological studies showing that intake of antibiotics in infancy increases the risk of being overweight at 7 years of age [147,148], and a recent study has shown that even prenatal exposure to antibiotics increases the risk of childhood obesity [149], perhaps through an effect on the maternal gut microbiota. Interestingly, although the risk of being overweight in children of mothers with normal pre-pregnancy body mass index (BMI) is increased by antibiotic treatment early in life, the opposite appears to be the case for children of overweight and obese mothers, for whom the risk is reduced [147]. In addition, non-randomized intervention studies have shown an increase in BMI in adults following antibiotic treatment, especially after *Helicobacter pylori* eradication in patients with gastric ulcer [150,151]. Increased content of SCFA and lower caloric output have been found in feces of mice treated with antibiotics, despite similar caloric intake as the non-treated mice [152], which support previous suggestions of an increased energy harvest leading to obesity [51]. In contrast to these findings, other mouse studies have shown improved glucose tolerance that was independent of weight changes, and also lower levels of circulating lipopolysaccharide and a lower bacterial count following antibiotic treatment, all indications of an improved metabolic state [153,154]. Recently it has been demonstrated that treatment of infant mice with antibiotics in subtherapeutic dosage increases adiposity, even later in life, and confers substantial compositional changes in the microbiota, which when transplanted to germ-free animals induces adiposity [152,155]. In humans, treatment with vancomycin has been shown to reduce peripheral insulin sensitivity in individuals with metabolic syndrome, whereas treatment with amoxicillin does not, reflecting the preferential targeting of Gram-positive butyrate-producing bacteria by the former [156]. Although current studies indicate that antibiotics have not only short- but also long-term effects [157] on the diversity and/or configuration of the gut microbiota, the possible role of antibiotics in development of human metabolic diseases needs further exploration.

### Bariatric surgery

Surgical weight-loss intervention, generally referred to as bariatric surgery, is currently the most effective treatment for morbid obesity. In addition to inducing and sustaining a substantial weight loss it is well established that bariatric surgery improves pancreatic beta-cell function through enhanced GLP-1 secretion, enhances insulin sensitivity, promptly ameliorates T2D and improves cardiovascular risk factors, thereby reducing mortality [158]. Recent studies suggest that the more long-term health benefits of bariatric surgery may in part be due to alterations in the gut microbiota. In non-obese rats, Roux-en-Y gastric bypass (RYGB) confers a dramatic shift in microbiota composition, with a 52-fold increase in Proteobacteria and reduction of Firmicutes and Bacteroidetes. These effects in rodents occur within 1 week of surgery and are apparently independent of the induced weight change and caloric restriction [159]. Studies in humans showing similar changes on the phylum level [14,160,161] in addition to increased overall richness substantiate the potent effect of surgery on the microbiota configuration. Studies showing correlation between specific bacterial genera and species (such as *Faecalibacterium prausnitzii*) and metabolic and inflammatory variables [14,15], as well as change in white adipose tissue gene expression [160], point to the potential role of microbiota as a mediator of the beneficial metabolic effects of bariatric surgery. Transmissibility of reduced adiposity through transplantation of gut microbiota from RYGB mice to germ-free mice further substantiates a potential causative effect [159].

### Transplantation - evidence of causality?

Spurred on by the safe and highly efficacious application of fecal microbiota transplantation in the treatment of recurrent *C. difficile* enterocolitis [162,163], transplantation of fecal matter has gained interest as a potential modulator of cardio-metabolic disorders. In a study of human twins discordant for obesity, colonization of germ-free mice with fecal microbiota from an obese donor induced weight gain, whereas stools from a lean twin did not [54]. Also, cohousing of coprophagic mice inoculated with either obese or lean microbiota protected the recipients of the former from developing the concurrent obese phenotype, although only when the mice were fed a high-fiber low-fat diet and not when fed high-fat, low-fiber diet [54]. A human study investigating 18 insulin resistant men who were randomized to duodenal infusion of either autologous fecal microbiota or microbiota from a heterologous lean donor showed improvement in insulin sensitivity 6 weeks after infusion of microbiota from lean donors (but not of autologous microbiota), along with increased microbiota diversity and an increase in butyrate-producing bacteria [164]. Recently it has been demonstrated that knock-out of MyD88, an adaptor molecule for Toll-like receptors, in mouse intestinal epithelial cells protects the animal from developing obesity and diabetes when fed a high fat diet, and knocking out the gene in obese animals induces weight loss [165].

Interestingly, transplanting gut microbiota from MyD88 knock-out mice fed a high-fat diet reduced weight gain and fat mass development in germ-free recipients fed a similarly high-fat diet. So even though data on any long-term effects are still needed, these pioneering studies in humans and rodents suggest that once a phenotype-specific gut microbiota is established in a given species, the phenotype in question can be transferred to another individual or species susceptible to the pathogenic impact of the given microbiota community.

## Concluding remarks

Although there is convincing evidence that dysbiotic states correlate with cardio-metabolic disorders, the underlying mechanisms have generally not been elucidated in depth. To exploit these and their potentially causal links to the cardiovascular health spectrum, future studies will probably concentrate on functional and mechanistic studies of potentially pathogenic gut microbiota in animal and cellular models, complemented by prospective observational and intervention studies of cardio-metabolic cases that are initially treatment-naïve to avoid confounding by various drugs. Much interest will be given to integrating gut microbiomics discoveries with findings from other pertinent high-throughput techniques such as genomics, epigenomics, metabolomics, lipidomics and peptidomics (of the host) and polymorphism metagenomics, meta-transcriptomics and meta-proteomics (of the microbiota).

Based on current knowledge, as discussed in this review, we hypothesize firstly that certain endogenous or exogenous stimuli (perhaps food, stress or infections) trigger a subclinical gut mucosal inflammation, which, depending on the genetic susceptibility of an individual, favor growth and expansion of a community of specific disease-promoting gut bacteria. Secondly, specific disease-promoting microbiota can be transmitted from an affected donor to a genetically susceptible recipient, where they reproduce the disease phenotype. Finally, it may be a general phenomenon in many disorders, including cardio-metabolic diseases, that once a specific pathological microbiota had developed it constitutes a self-perpetuating pathogenic risk factor. Although ground-breaking research in recent years has enormously expanded our understanding of the microbiota-host interplay, the numerous cellular and molecular mechanisms involved in the pathophysiological interactions await further discovery and characterization.

## Box 1: Gut microbiota research methodologies

### 16S rRNA-based profiling

16S rRNA gene profiling involves targeted amplification and subsequent next-generation sequencing of phylogenetically informative marker sequences of microbial DNA [20]. The taxonomic level of information varies according to the subsequent applied technology: denaturing gradient gel electrophoresis (DGGE), temperature gradient gel electrophoresis (TGGE) and terminal restriction fragment length polymorphism (T-RFLP) give poor taxonomic information, whereas phylogenic microarrays (such as HitChip), 454 pyrosequencing and Illumina MiSeq/HiSeq platforms provide quantitative information and extended taxonomic identification [21,22].

From 16S rRNA data the number of different taxa present in a sample and their relative abundance can be estimated by comparisons with reference-sequence databases or by using software packages to cluster highly similar sequences into OTUs [21]. Thus, targeted 16S rRNA sequencing can describe the bacterial community in terms of which taxa or OTUs are present, their relative abundance, and their phylogenetic relationships [22]. In the literature on 16S rRNA-derived taxa the term phylotype is often used instead of species, as this method of taxonomic classification is not based on phenotypic characteristics of the microbe but on phylogenetic analysis of 16S rRNA sequences. About 3 million aligned and annotated 16S rRNA sequences are currently available as part of the Ribosomal Database Project [23].

### Shotgun sequencing (quantitative metagenomics)

In contrast to the targeted 16S rRNA approach, the untargeted metagenomics approach involves direct next-generation sequencing of community DNA without amplification of specific regions. By in-depth sequencing the whole genomic content of the gut microbiota rather than marker genes alone, a less biased and higher-resolution view of the bacterial composition can be acquired through comparison with bacterial reference genomes and microbial gene catalogs [24] or through *de novo* assembly [25]. In addition, information can be gained about additional elements of the microbial community, including fungi and viruses. The 2014 metagenomic catalog of the human gut microbiome (consisting of 1,267 individual metagenomes compiled from the Metagenomics of the Human Intestinal Tract (MetaHIT) consortium, Chinese and Human Microbiome Project studies) includes up to 9.9 million non-redundant genes [26]. In addition to benefits in terms of improved taxonomic description, shotgun metagenomic sequencing enables a more detailed assessment of the functional potential of the microbial community through comparison with databases of metabolic pathways (such as the Kyoto Encyclopedia of Genes and Genomes) [21]. The overwhelming amount of shotgun sequencing-based metagenomics data requires a continued development and improvement of bioinformatics tools to enhance the analysis and understanding of the complexity of the human gut microbiome. This need is clearly illustrated by the fact that currently only a minority of the gut microbial genes can be annotated to known genomes of cultured microbes. Therefore, advanced bioinformatics methods based on co-abundance have been developed to enable grouping of microbial genes into 741 large MetaGenomic Units (MGUs) representing as-yet uncultured bacterial species. Characteristically, each MGU contains more than 700 microbial genes [25]. An example of a quantitative metagenomics pipeline is given in Figure 1.

## Additional file

Additional file 1: Table S1. Human clinical studies of trimethylamine-containing compounds in relation to cardio-metabolic disorders. **Table S2 in Additional file 1:** Human clinical trials of probiotic supplements in relation to cardio-metabolic traits.

## Abbreviations

BMI: Body mass index
CVD: Cardiovascular diseases
GLP: Glucagon-like peptide
OTU: Operational taxonomic unit
PC: Phosphatidylcholine
PYY: Peptide YY
RYGB: Roux-en-Y gastric bypass
SCFA: Short chain fatty acids
T2D: Type 2 diabetes
TMAO: Trimethylamine-*N*-oxide
