# Supplementary material for: The gut microbiome in cardio-metabolic health
Source: Genome Med. 2015 Mar 31;7(1):33. doi: 10.1186/s13073-015-0157-z (PMC4378584; doi:10.1186/s13073-015-0157-z)
Supplement: Additional file 1: Table S1. — Human clinical studies of trimethylamine-containing compounds in relation to cardio-metabolic disorders. Table S2 in Additional file 1: Human clinical trials of probiotic supplements in relation to cardio-metabolic traits. [file 13073_2015_157_MOESM1_ESM.docx]

# Supplementary tables

**Supplementary table 1. Human clinical studies of trimethylamine-containing compounds in relation to cardio-metabolic disorders**

| **Study** | **Participants** | **Main findings** |
| --- | --- | --- |
| Danne et al. (2007) [1] | 217 patients with suspected ACS | - Choline was a significant and independent predictor of MACE during 30-days of follow-up. |
| LeLeiko et al. (2009) [2] | 108 patients presenting with chest pain | - Choline levels predicted 30-day cardiac events (i.e. nonfatal myocardial infarction, congestive heart failure, need for revascularization, and death). |
| Wang et al. (2011) [3] | 1,876 patients undergoing elective cardiac evaluation | - Choline, betaine and TMAO showed dose-dependent associations with prevalent peripheral artery disease, coronary artery disease, and history of myocardial infarction, as well as overall CVD. |
| Tang et al. (2013) [4] | 4,007 patients undergoing elective diagnostic cardiac catheterization | - Increased levels of TMAO were associated with an increased risk of a MACE, and an elevated TMAO level was an independent predictor of MACE during 3 years of follow-up. - Elevated level of TMAO predictive of MACE even in low-risk subgroups. |
| Koeth et al. (2013) [5] | 2,595 patients undergoing elective cardiac evaluation | - Signifi­cant dose-dependent associations between carnitine concentration and risks of prevalent coronary artery disease, peripheral artery disease and overall CVD, also when adjusted for traditional CVD risk factors. - Elevated carnitine concentration was an independent predictor of MACE during 3-years of follow-up. - Significant association between carnitine concentration and incident cardiovascular event risk, but only among those participants with concurrent high plasma TMAO concen­trations. |
| Wang et al. (2014) [6] | 3,903 patients undergoing elective diagnostic coronary angiography | - Higher plasma choline and betaine levels were associated with a 1.9-fold and 1.4-fold increased risk of MACE, respectively. - Elevated choline and betaine concentration was an independent predictor of MACE during 3-years of follow-up. - Significant association between choline and betaine concentration and incident cardiovascular event risk, but only among those participants with concurrent high plasma TMAO concen­trations. |
| Tang et al. (2014) [7] | 720 patients with stable HF | - Modest but significant correlation between TMAO concentrations and BNP levels. - Higher plasma TMAO levels were associated with a 3.4-fold increase in 5-year mortality risk and remained predictive when adjusted for traditional risk factors and BNP levels. |
| Lever et al. (2014) [8] | 475 patients with ACS | - In participants with T2D (n = 79), high plasma betaine was associated with increased frequency of heart failure and all cardiovascular events and high TMAO was a marker of all outcomes (death, myocardial infarction, heart failure, unstable angina, and all cardiovascular events). - In participants without diabetes (n = 396), low plasma betaine was associated with secondary myocardial infarction, unstable angina, and all cardiovascular events and TMAO was only significant for death and heart failure. |
| Trøseid et al. (2014) [9] | 155 patients with chronic HF | - Plasma levels of TMAO, choline, and betaine were elevated compared to control participants, with the highest levels in patients with NYHA class III and IV. - TMAO, but not choline or betaine, was associated with reduced transplant-free survival during 5.2 years of follow-up. |
| Tang et al. (2015) [10] | 112 patients with chronic systolic HF | - TMAO levels were significantly higher in patients with diabetes mellitus and in patients with NYHA class III or greater. - Elevated TMAO, choline, and betaine levels were associated with higher NT-proBNP levels and more advanced left ventricular diastolic dysfunction, but not systolic dysfunction or inflammatory and endothelial biomarkers. - Higher choline, betaine, and TMAO predicted increased risk for 5-year adverse clinical events (i.e. death, transplantation), but only TMAO did so independently of other risk factors. |
| Tang et al. (2015) [11] | 521 patients with and 3,166 without CKD | - TMAO level among CKD participants was markedly higher than in non-CKD participants. - Within CKD participants, higher (fourth versus first quartile) TMAO level was associated with a 2.8-fold increased mortality risk and elevated TMAO levels was an independent predictor of 5-year mortality risk. |
| Abbreviations:  ACS, acute coronary syndrome; BNP, brain natriurectic peptide; CKD, chronic kidney disease; CVD, cardio-vascular disease; HF, heart failure; MACE, major adverse cardiac event (death, myocardial infarction, stroke or revascularization); NT-proBNP, N-terminal pro brain natriuretic peptide; NYHA, New York Heart Association runctional classification of heart failure | | |

**Supplementary table 2. Human clinical trials of probiotic supplements in relation to cardio-metabolic traits**

| **Study** | **Participants** | **Design** | **Probiotic** | **Intervention effects** |
| --- | --- | --- | --- | --- |
| De Roos et al.  (1999) [12] | 78 ♀/♂  Healthy | Randomized, placebo-controlled, | *L. acidophilus* L-1 | ↔ TC, LDL-C, HDL-C, TAG |
| Greany et al.  (2007) [13] | 55 ♀/♂  Healthy | Randomized, placebo-controlled, single-blinded | *L. acidophilus* DDS-1*, B. longum* UABL-14 | ↔ TC, LDL-C, HDL-C, TAG |
| Ataie-Jafari et al.  (2009) [14] | 14 ♀/♂  Dyslipidemia | Randomized, cross-over | *L. acidophilus, B. lactis* | ↔ LDL-C, HDL-C, TAG, LDL-C/HDL-C  ↓ TC |
| Andreasen et al. (2010) [15] | 45 ♂  T2D,IGT,NGT | Randomized, placebo-controlled, double-blinded | *L. acidophilus* NCFM | ↑ Insulin sensitivity (due to increased insulin resistance in the placebo group)  ↔ TNF, IL-6, IL-1RA |
| Asemi et al.  (2010-13) [16-19] | 70 ♀  Pregnant | Randomized, placebo-controlled, single-blinded | *L. acidophilus* LA5, *B. animalis* BB12 | ↑ glutathione reductase  ↔ TNFα, TC, LDL-C, HDL-C, TAG, TC/HDL-C, FPG,  ↔ SBP, DBP  ↓ hsCRP, insulin, HOMA-IR |
| Kadooka et al.  (2013) [20] | 14 ♀/♂  Central obesity | Randomized, placebo-controlled, double-blinded | *L. gasseri* SBT2055 | ↓ Visceral adiposity, BMI, waist and hip circumferences, body fat % |
| Ejtahed et al.  (2011) [21] | 60 ♀/♂  T2D | Randomized, placebo-controlled, double-blinded | *L. acidophilus* LA5, *B. animalis* BB12 | ↓ TC, LDL-C, TC/HDL-C, LDL-C/HDL-C |
| Ejtahed et al.  (2012) [22] | 64 ♀/♂  T2D | Randomized, placebo-controlled, double-blinded | *L. acidophilus* LA5, *B. animalis* BB12 | ↓ FPG, HbA1c  ↑ glutathione peroxidase, superoxide dismutase, total antioxidant status |
| Luoto et al.  (2012) [23] | 256 ♀  Pregnant | Randomized, placebo-controlled, double-blinded | *L. rhamnosus, B. lactis* | ↑ Adiponectin in colostrum |
| Moroti et al.  (2012) [24] | 20 ♀  T2D | Randomized, placebo-controlled, double-blinded | *L. acidophilus*, *B. bifidum* | ↑ HDL-C  ↓ FPG |
| Jung et al.  (2013) [25] | 64 ♀/♂  Overweight/Obesity | Randomized, placebo-controlled, double-blinded | *L. gasseri* BNR17 | ↔ FPG, insulin, HbA1c,TC, LDL-C, HDL-C, TAG  ↔ Visceral adiposity, BMI, waist and hip circumferences, body fat % |
| Mazloom et al. (2013) [26] | 34 ♀/♂  T2D | Randomized, placebo-controlled, single-blinded | *L. acidophilus, L. bulgaricus, L. bifidum, L. casei* | ↔ TC, LDL-C, HDL-C, TAG, FPG, insulin, malondialdehyde, hsCRP, IL-6, HOMA-IR |
| Abbreviations: ♂, male;♀, female; BMI, body mass index; DBP, diastolic blood pressure; FPG, fasting plasma glucose; HbA1C, glycosylated hemoglobin A1c; HDL-C, high-density lipoprotein cholesterol; HOMA-IR, homeostatic model assessment of insulin resistance; hsCRP, high-sensitivity C-reactive protein; IGT, impaired glucose tolerance; IL-1RA, interleukin-1 receptor antagonist; IL-6, interleukin-6; LDL-C, low-density lipoprotein cholesterol; NGT, normal glucose tolerance; SBP, systolic blood pressure; T2D, type 2 diabetes; TAG, triacylglycerol; TC, total cholesterol. | | | | |

# References

1. Danne O, Lueders C, Storm C, Frei U, Möckel M. Whole blood choline and plasma choline in acute coronary syndromes: Prognostic and pathophysiological implications. Clinica Chimica Acta. 2007;383:103-9.

2. LeLeiko RM, Vaccari CS, Sola S, Merchant N, Nagamia SH, Thoenes M, et al. Usefulness of elevations in serum choline and free f2-isoprostane to predict 30-day cardiovascular outcomes in patients with acute coronary syndrome. Am J Cardiol. 2009;104:638-43.

3. Wang Z, Klipfell E, Bennett BJ, Koeth R, Levison BS, DuGar B, et al.: Gut flora metabolism of phosphatidylcholine promotes cardiovascular disease. Nature. 2011;472:57-63.

4. Tang WHW, Wang Z, Levison BS, Koeth RA, Britt EB, Fu X, et al. Intestinal microbial metabolism of phosphatidylcholine and cardiovascular Risk. N Engl J Med. 2013;368:1575-84.

5. Koeth RA, Wang Z, Levison BS, Buffa JA, Org E, Sheehy BT, et al.: Intestinal microbiota metabolism of l-carnitine, a nutrient in red meat, promotes atherosclerosis. Nat Med. 2013;19:576-85.

6. Wang Z, Tang WHW, Buffa JA, Fu X, Britt EB, Koeth RA, et al. Prognostic value of choline and betaine depends on intestinal microbiota-generated metabolite trimethylamine-N-oxide. Eur Heart J. 2014;35:904-10.

7. Tang WHW, Wang Z, Fan Y, Levison B, Hazen JE, Donahue LM, et al. Prognostic value of elevated levels of intestinal microbe-generated metabolite trimethylamine-N-oxide in patients with heart failure: refining the gut hypothesis. J Am Coll Cardiol. 2014;64:1908-14.

8. Lever M, George PM, Slow S, Bellamy D, Young JM, Ho M, et al.: Betaine and trimethylamine-N-oxide as predictors of cardiovascular outcomes show different patterns in diabetes mellitus: an observational study. PLOS ONE. 2014;9:e114969.

9. Troseid M, Ueland T, Hov JR, Svardal A, Gregersen I, Dahl CP, et al.: Microbiota-dependent metabolite trimethylamine-N-oxide is associated with disease severity and survival of patients with chronic heart failure. J Intern Med. 2014, doi: 10.1111/joim.12328.

10. Tang WHW, Wang Z, Shrestha K, Borowski AG, Wu Y, Troughton RW, et al. Intestinal microbiota-dependent phosphatidylcholine metabolites, diastolic dysfunction, and adverse clinical outcomes in chronic systolic heart failure. J Card Fail. 2015;21:91-6.

11. Tang WH, Wang Z, Kennedy DJ, Wu Y, Buffa JA, Agatisa-Boyle B, et al. Gut microbiota-dependent trimethylamine N-oxide (TMAO) pathway contributes to both development of renal insufficiency and mortality risk in chronic kidney disease. Circ Res. 2015;116:448-55.

12. de Roos NM, Schouten G, Katan MB. Yoghurt enriched with Lactobacillus acidophilus does not lower blood lipids in healthy men and women with normal to borderline high serum cholesterol levels. Eur J Clin Nutr. 1999;53:277-80.

13. Greany KA, Bonorden MJL, Hamilton-Reeves JM, McMullen MH, Wangen KE, Phipps WR, et al. Probiotic capsules do not lower plasma lipids in young women and men. Eur J Clin Nutr. 2007;62:232-7.

14. Ataie-Jafari A, Larijani B, Alavi Majd H, Tahbaz F. Cholesterol-lowering effect of probiotic yogurt in comparison with ordinary yogurt in mildly to moderately hypercholesterolemic subjects. Ann Nutr Metab. 2009;54:22-7.

15. Andreasen A, Larsen N, Pedersen-Skovsgaard T, Berg R, Moller K, Svendsen K, et al. Effects of Lactobacillus acidophilus NCFM on insulin sensitivity and the systemic inflammatory response in human subjects. Br J Nutr. 2010, 104:1831-8.

16. Asemi Z, Jazayeri S, Najafi M, Samimi M, Mofid V, Shidfar F, et al. Effects of daily consumption of probiotic yoghurt on inflammatory factors in pregnant women: a randomized controlled trial. Pak J Biol Sci. 2011;14:476-82.

17. Asemi Z, Samimi M, Tabasi Z, Talebian P, Azarbad Z, Hydarzadeh Z, et al. Effect of daily consumption of probiotic yoghurt on lipid profiles in pregnant women: a randomized controlled clinical trial. J Matern Fetal Neonatal Med. 2012; 25:1552-6.

18. Asemi Z, Samimi M, Tabassi Z, Naghibi Rad M, Rahimi Foroushani A, Khorammian H, et al. Effect of daily consumption of probiotic yoghurt on insulin resistance in pregnant women: a randomized controlled trial. Eur J Clin Nutr. 2013;67:71-4.

19. Asemi Z, Jazayeri S, Najafi M, Samimi M, Mofid V, Shidfar F, et al. Effect of daily consumption of probiotic yogurt on oxidative stress in pregnant women: a randomized controlled clinical trial. Ann Nutr Metab. 2012;60:62-8.

20. Kadooka Y, Sato M, Ogawa A, Miyoshi M, Uenishi H, Ogawa H, et al. Effect of Lactobacillus gasseri SBT2055 in fermented milk on abdominal adiposity in adults in a randomised controlled trial. Br J Nutr. 2013;110:1696-1703.

21. Ejtahed HS, Mohtadi-Nia J, Homayouni-Rad A, Niafar M, Asghari-Jafarabadi M, Mofid V, et al. Effect of probiotic yogurt containing Lactobacillus acidophilus and Bifidobacterium lactis on lipid profile in individuals with type 2 diabetes mellitus. Jo Dairy Sci. 2011;94:3288-94.

22. Ejtahed H, Mohtadi-Nia J, Homayouni-Rad A, Niafar M, Asghari-Jafarabadi M, Mofid V. Probiotic yogurt improves antioxidant status in type 2 diabetic patients. Nutrition. 2012;28:539-43.

23. Luoto R, Laitinen K, Nermes M, Isolauri E. Impact of maternal probiotic-supplemented dietary counseling during pregnancy on colostrum adiponectin concentration: a prospective, randomized, placebo-controlled study. Early Hum Dev. 2012;88:339-44.

24. Moroti C, Souza Magri LF, de Rezende Costa M, Cavallini DC, Sivieri K. Effect of the consumption of a new symbiotic shake on glycemia and cholesterol levels in elderly people with type 2 diabetes mellitus. Lipids Health Dis. 2012;11:29.

25. Jung SP, Lee KM, Kang JH, Yun SI, Park HO, Moon Y, et al. Effect of Lactobacillus gasseri BNR17 on Overweight and Obese Adults: A Randomized, Double-Blind Clinical Trial. Korean J Fam Med. 2013;34:80-9.

26. Mazloom Z, Yousefinejad A, Dabbaghmanesh MH. Effect of probiotics on lipid profile, glycemic control, insulin action, oxidative stress, and inflammatory markers in patients with type 2 diabetes: a clinical trial. Iran J Med Sci. 2013;38:38-43.
